# Construction of a middle-range nursing theory for transpersonal home care

**DOI:** 10.1590/0034-7167-2024-0200

**Published:** 2025-03-10

**Authors:** Luana Tonin, Maria Ribeiro Lacerda, Paula Manuela Jorge Diogo, Marcos Antônio Gomes Brandão, Marilene Loewen Wall, Paulo Roberto Lima Falcão do Vale

**Affiliations:** I Pontíficia Universidade Católica do Paraná. Curitiba, Paraná, Brazil; II Universidade Federal do Paraná. Curitiba, Paraná, Brazil; III Escola Superior de Enfermagem de Lisboa. Lisboa, Portugal; IV Universidade Federal do Rio de Janeiro. Rio de Janeiro, Rio de Janeiro, Brazil; V Universidade Federal do Recôncavo da Bahia. Santo Antônio de Jesus, Bahia, Brazil

**Keywords:** Nursing Theory, Concept Formation, Home Health Nursing, Home Care Services, Nursing Care, Teoría de Enfermería, Formación de Concepto, Cuidados de Enfermería en el Hogar, Servicios de Atención de Salud a Domicilio, Atención de Enfermería

## Abstract

**Objectives:**

to describe the development of a middle-range nursing theory for transpersonal home care.

**Methods:**

this is a theoretical study that used concept analysis, statement synthesis and derivation. It combined theoretical deduction strategies with induction, based on literature review and concept analysis.

**Results:**

the central concept was derived from Jean Watson’s Unitary Care Science theoretical framework. The synthesis of the statements between the concepts led to the elaboration of a middle-range theory. Fourteen assumptions and ten propositions were constructed, in addition to a diagram representing the middle-range theory.

**Final Considerations:**

the constructed theory is intended to contribute to home care, covering physiological, psychological, sociocultural, developmental and spiritual needs. Theoretical and empirical validation is suggested to confirm, refute or renew the theory concepts, assumptions and propositions.

## RESUMEN

**Objetivos:**

describir el desarrollo de una teoría de enfermería de rango medio para el cuidado transpersonal domiciliario.

**Métodos:**

se trata de un estudio teórico, que utilizó análisis de conceptos, síntesis y derivación de enunciados. Combinó estrategias de deducción teórica con inducción, basadas en revisión de literatura y análisis de conceptos.

**Resultados:**

el concepto central se derivó del marco teórico de la Ciencia del Cuidado Unitario de Jean Watson. La síntesis de enunciados entre conceptos condujo a la elaboración de la teoría de rango medio. Se construyeron 14 supuestos y diez proposiciones, además de un diagrama que representa la teoría de rango medio.

**Consideraciones Finales:**

la teoría construida pretende contribuir al cuidado domiciliario, cubriendo necesidades fisiológicas, psicológicas, socioculturales, de desarrollo y espirituales. Se sugiere validación teórica y empírica para confirmar, refutar o renovar los conceptos, supuestos y proposiciones de la teoría.

## INTRODUCTION

This study proposes the construction of a middle-range nursing theory focused on transpersonal home care. In line with the “Ministry of Health Research Priorities Agenda”, in the area of health programs and policies, especially with regard to home care^(1)^, this research aligns with the priorities identified by home nursing leaders in several countries^(2)^.

The projected increase in demand for home care in the coming decades, driven by population aging and global demographic changes, makes it essential to develop evidence-based guidelines and restructure healthcare delivery systems^(3)^. In contexts such as the European Union and the United States of America, there is a growing need for home care, reflecting the evolution of care practices and the impact on the population’s health conditions^(4,5)^.

The COVID-19 pandemic has further intensified the importance of home care as a response to global epidemiological, social and economic challenges^(6,7)^. The effectiveness of home care transcends the conventional health-disease approach, incorporating daily, cultural and contextual elements^(8,9,10)^.

Jean Watson’s Theory of Human Caring/Unitary Caring Science is notable for involving a unique approach, emphasizing the importance of lived experience, recognizing the mind-body-spirit dimensions as an energetic field belonging to the unitary universe^(11)^.

Influenced by humanitarian and existentialist principles, this theory includes transpersonal care, the moment of care and elements of the Clinical Caritas Veritas process^(11)^. Valuing multiple ways of knowing, the theory helps nurses in structuring practice and in the relationship with the person being cared for^(12)^.

In the home care approach, the importance of authenticity and a relationship of trust is highlighted, honoring the integrity and dignity of individuals and families^(11)^. Nurses’ authentic presence allows for a meaningful connection, facilitating the understanding of needs and promoting transpersonal home care.

Reflecting on the nursing discipline, the use of theories contributes to technical-scientific knowledge and critical thinking^(12)^. The importance of continuous development of knowledge is highlighted, with middle-range theories, indicating progress in the epistemology of the area^(13)^.

For Watson^(14)^, Meleis^(15)^, Smith and Liehr^(13)^, middle-range, long-range and situation-specific theories provide an evolved, shared and unitary vision of the world of health. Each middle-range theory reflects philosophies that guide abstract views, influencing the meaning of the theory^(13)^.

Walker and Avant^(16)^ describe the levels of theory as metatheory, grand theory, middle-range theory, and practical theory. Grand theories, such as the Theory of Human Caring/Unitary Caring Science^(11)^, are frameworks that explain abstract phenomena, whereas middle-range theories are more circumscribed, applying to specific contexts, such as home care^(13)^.

Considering the above, investigating home care and proposing theoretical relationships among the elements and concepts that bring together the phenomenon can represent a contribution to nursing. This is because the home context and care need to be discussed continuously, as this environment is permeated by instabilities in physiological, psychological, sociocultural, developmental and spiritual dimensions, consequently presenting regional and territorial variances.

In order to elucidate the relevance of this study, it is highlighted that investigations that address aspects related to home care and interfaces with theoretical frameworks are necessary and current. Another factor to highlight among the arguments that support this study concerns gaps in scientific production in relation to the subject. In extensive integrative reviews carried out on middle-range theories, none of them address the specificities of home care^(17,18)^.

Thus, it was assumed that transpersonal care is relevant for care provided at home, in the same way as the Theory of Human Caring/Science of Unitary Caring, a great theory to be used to deduce a middle-range theory for home care.

## OBJECTIVES

To describe the development of a middle-range nursing theory for transpersonal home care.

## METHODS

### Ethical aspects

The research, although theoretical, respected the copyright of included publications. It followed Law 9610/1998, which regulates copyright, considering the offering of a scientific work to the public with the author’s consent as a publication.

### Study design

This is a theoretical study, descriptive-exploratory in nature, with a qualitative approach. In the development of middle-range theory, strategies can follow an inductive or deductive orientation or combine both. Deductive strategies are based on grand theories or theoretical models, whereas the inductive approach starts from the data to organize and build a new theory^(19)^.

The strategies proposed by Walker and Avant^(16)^ were adopted in this study, including analysis, synthesis and derivation. The choice of this strategy aims at the primarily deductive construction of a middle-range theory for home care, allowing, in this case, the theoretical deduction (derivation) of a great nursing theory (Theory of Human Caring/Science of Unitary Care)^(11)^.

### Methodological procedures

#### Data collection, organization and analysis

In the first stage of middle-range theory development, concept analysis occurred, comprised of eight sequential stages, which include concept selection, determination of analysis objectives, identification of uses of the chosen concept, determination of defining attributes, identification of a case model, identification of additional cases, identification of antecedents and consequences, and definition of empirical indicators^(16)^.

The initial stage of the concept analysis process focused on the concept of home care. The choice of this concept is justified by the researchers’ concerns. Moreover, there is confusion in the literature about related terms, such as home care and home assistance.

The next phase involved identifying all uses of the concept, guided by the following question: what is the definition of the concept of home care for nursing?

The selected databases included BDENF, LILACS, PubMed, Science Direct, Oasis Br and CINAHL. For each search, the following strategy was used: (“Home care services” OR “Home Health Nursing” OR “Home Nursing”) AND “Nursing Care”. The search was guided by an experienced librarian.

Studies from a five-year period were considered. The results from the search strategy used were imported into the EndNote Web® reference manager. The pre-selection of primary studies was performed by reading titles and abstracts, with subsequent selection for reading in full. Both stages were performed by two researchers, independently and blindly, in order to reduce study selection bias, and disagreements during the selection process were discussed. Studies that were repeated and that did not correspond to the review’s guiding question were excluded.

A total of 59 studies were included in concept analysis. These studies were thoroughly examined, synthesized and used to compose the phenomenon analysis, identifying related concepts and supporting the synthesis of statements and theoretical derivation.

Concept analysis, by generating extensive material, can compromise the concept’s internal consistency^(16)^. To overcome this, QSR NVivo 10 software was used for data organization and analysis^(20)^.

After concept selection, goal determination, and use identification, subsequent stages included importing files into NVivo 10. Reading the selected material was accompanied by the creation of memos and nodes, essential for reflection and further analysis^(20)^.

The stage to determine the definitional attributes, also called critical attributes, involves identifying words or expressions frequently used by authors to describe the characteristics of the concept of interest^(16)^. The guiding question was: what attributes or characteristics indicated in the literature are present in the concept of home care? The selected words and expressions were organized into a corresponding node.

In the model and additional case studies stage, the objective is to illustrate the concept under study through an example, real or fictitious, that contains its defining attributes. It should help to elucidate what the concept exactly is and what it cannot be^(16)^.

The situations, events or phenomena that precede the concept of interest are considered antecedents^(16)^. In order to find the antecedents of the concept under analysis, the following question was established: what events precede the existence of the concept of home care?

The consequences of this concept are events or situations resulting from its use. They may or may not coincide with the defining attributes^(16)^. To verify the presence of consequences related to the concept of home care, the question was used: what resulted after applying the concept of home care?

In the final stage of concept analysis, we sought to identify empirical references for the defining attributes of the concept. Empirical references are categories or classes of observable phenomena related to defining attributes^(16)^. The selected literature was (re)consulted to determine whether empirical indicators of home care were identified and whether corresponding instruments or measures exist.

In the second stage of middle-range theory construction^(16)^, synthesis of statements of concepts, synthesis was carried out through specifications of the relationships based on evidence from selected studies and statements combined with the Theory of Human Caring/Science of Unitary Care theoretical framework^(11)^.

In theoretical derivation (the third stage), a theorist creates a new theory by using analogy with an existing theory. Reasoning by analogy is fundamental to innovation, allowing the transfer of ideas from one field to another. The theorist seeks insight from related fields and redefines conceptual frameworks to form a unique theory^(16)^. In this case, structural aspects of the Theory of Human Caring/Unitary Caring Science were derived^(11)^.

Concerning the classification in terms of objectives^(15)^, it is argued that the middle-range theory for transpersonal home care constructed can be considered descriptive, because it focuses on describing the phenomena related to healthcare in this environment. It examines the properties, circumstances and patterns that occur in home care, helping to categorize and classify the observations made in this context. Although it may contain predictive elements, its main function is explanatory, providing a basis for understanding how and why certain phenomena occur in home care and what their possible consequences are.

## RESULTS

When characterizing the studies analyzed, it was identified that 33 of studies originated from South American countries, but geographic distribution was quite varied, also containing studies from Europe (14), Oceania (1), Asia (5) and North America (4). Two studies were developed between partnerships between two countries (Portugal and Brazil), which means that the topic is of interest to several places in the world.

Regarding the professional category, 50 were carried out by nurses as main authors, professionals from the medical category (seven), and also a geographer and an occupational therapist. As for the year of publication of analyzed studies, the time frame established was between January 2015 and December 2019, with 11 dating from 2015, 15 from 2016, 12 from 2017, December from 2018, and 11 from 2019.

The concept of home care varies in meaning among countries, being practiced by nurses in some places and predominantly by family members in others. It is considered an alternative modality to the hospital for continuity and/or cost reduction. The practice faces challenges, requiring an understanding of the environment, covering economic, social, emotional and ethical aspects. Moreover, it stands out as a modality that is closer and oriented to users’ needs. Home care involves an intimate space of people, promoting a unique transpersonal interrelationship among healthcare professionals, the person being cared for and the family. Practiced in the place of residence, it is dynamic, unique and based on each patient’s and family’s needs. The presence of a social support network, social and economic aspects, family participation and effective relationships among professionals, patients and family are crucial. The development of a model case exemplifies the attributes^(21)^.

The opposite case demonstrates the opposite of what is defined as home care, which does not demonstrate good communication among patient, family and health team, not indicating the planning of home nurses’ actions, with a focus on physical, social, cultural and spiritual aspects^(21)^.

Home care is preceded by the need arising from conditions of illness, chronic diseases, functional disabilities, aging, and may be a preference of patients and family members. It requires emotional support and adjustments to the home structure, financial resources and adequate information.

The consequences include continuity of care, management of hospital beds, and reduction of complications and infections. In addition to this, it presents challenges and concerns, such as changes in home structure and transfer of costs, causing stress and affecting family organization. It impacts healthcare professionals and effective interrelationships among professionals, patients, and families, generating reflective learning.

Empirical indicators in the context of home care represent observable categories that demonstrate the occurrence of the concept. These indicators measure professional preparation prior to care, highlighting professional training and qualification, and record the transformation of professionals during care provision.

The participation of a social support network, whether formal or informal, is essential for the success of home care. Effective relationships between caregivers and those being cared for occur through the promotion of strengthening beliefs and emotional care, meeting physical, mental, social and spiritual needs.

### Middle-range nursing theory for transpersonal home care

Based on the assessment of the attributes, antecedents and consequences described in this study and the framework adopted to derive this middle-range theory, the following definition for home care was developed: care provided in home environments by nursing and an interprofessional team, from birth to terminality, which includes the interconnection between caregivers and persons being cared for, using the care process with a view to healing. It requires recognizing the peculiarities of the home context, the family and the social support network with planning, knowledge, technical-scientific skills, and respect for individual beliefs, culture and values.

After defining home care, based on the integrative review of the literature developed, other concepts related to the phenomenon were also extracted, such as nursing metaparadigms (health, human being, environment, nursing), caregiver, family, home context, management, interprofessional team, ethics in home care, social support network, home patient safety, professional training and transpersonal care. These concepts, when related, contribute to describe home care.

The affirmative synthesis procedure supported the elaboration of 14 assumptions and ten propositions, presented below:

Assumptions: home care is present throughout life cycle; the environment in which care is developed is the home, which has specificities of each structure, culture and context; when providing care in this environment, nurses guarantee the autonomy of the other (human being, caregiver and family) by respecting their free and responsible decisions, which is fundamental in the exercise of care; for transpersonal home care to occur, it is necessary to look for the circumstances of the other (human being, family and support network) (in other words, it means understanding the keys to the home context to understand how they act); consideration of social and spiritual factors, values, home context, beliefs and ideals, which surround human existence, will be necessary to immerse oneself in the circumstances of human beings and observe how they are affected; when providing transpersonal home care, the preservation of the identity of the other is created, i.e., caring for a subject of rights, a unique being in history, who has an identity carved in time, which caregivers must know how to respect and promote as much as possible; in the process of providing care at home, the needs experienced by the human being are alleviated, not only those of a physical nature, but also those of a psychological, social and spiritual nature (for this, an adequate capacity to listen to or receive the other will be necessary as well as adequate professional competency to resolve these needs); in the act of transpersonal home care, the preparation of a person providing care is fundamental (caring is only possible if we imagine what may happen in the future and what needs will arise, in order to respond with commitment and seriousness to these needs and avoid complications); adequately care for others, the person providing care must feel properly cared for (family caregiver and professional), and, to this end, self-care is the condition for the possibility of caring for others; accompanying the human being in a transpersonal way also means being attentive to mental health, understood not only as the absence of psychic pathologies, but also as the appropriation of one’s own cognitions, ideas, theories, paradigms and ways of understanding reality; in addition, support in transpersonal home care can also be offered to family caregivers and the social support network, through responsible case management, recognizing them, welcoming them, integrating them and strengthening their energy in care; accompanying the human being, family caregiver and social support network in a transpersonal way also means being attentive to the spiritual domain, through the awareness of being transcendent, knowledge of one’s own values and respect for the diversity of cultures and religions; competency for transpersonal home care is based on training at a theoretical level, on the formation of practical skills and abilities, on the moral capacity to be sensitive to suffering and to pay attention to others; to provide transpersonal care, one must rescue the integral vision of the being cared for, going against the contemporary mentality that follows the path of fragmentation and overspecialization.

Propositions of middle-range nursing theory for transpersonal home care: transpersonal care at home is a unitary relational care that illuminates the nurse-person relationship and the healing environment; when providing transpersonal care at home, attention is given to the body, mind and spirit; transpersonal nursing care at home is a special way of being, knowing and doing, with the aim of protecting, valuing and preserving human dignity; it implies commitment from the nursing professional in care, attending to more than physical needs and encompassing the person being cared for in their psychological, social and spiritual needs; only by establishing an adequate relationship of trust with the person being cared for and the family caregiver will we be able to make them share the information they consider relevant about the aspects that concern them in the home context, with the aim of having more information and tools to help them; to gain in-depth knowledge of aspects related to the higher-order needs of the person being cared for, it will be necessary to consider the individual’s social and cultural aspects and delve into their life history, considering all aspects that may affect them; Responsibility and professional collaboration are considered inseparable attitudes, since adequate professional practice implies collaboration and teamwork, in order to, in this case, obtain different professional visions to help the person being cared for and their family to meet their higher and lower order needs; it is considered that, in order to care for others, we must first be attentive to our spirituality, know it and work on it beforehand, and from our own experience as spiritual beings, we can be more attentive to the needs of this field in the person being cared for; communication skills and caring attitude, showing an active attitude, reducing physical and non-physical discomfort and promoting spiritual well-being; adequate communication skills will allow us to explore and detect early on the spiritual needs and resources of the person being cared for; the awareness of transpersonal care in the home will transcend time, space and physicality, becoming part of the complex energetic pattern of a person’s life.

**Figure 1:**
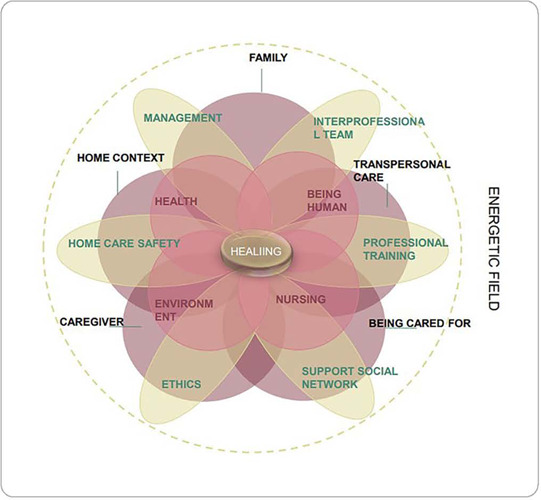
Representative diagram of middle-range nursing theory for transpersonal home care, Curitiba, Paraná, Brazil, 2021

After the concept of home care was analyzed and relational statements were synthesized between the concepts and the same derived from the basic theoretical framework, theory modeling was then carried out and will be presented below (Figure 1).

Applying the analogy, the diagram presented in the figure configures the middle-range nursing theory for transpersonal home care. It is understood that home care occurs in a variety of environments and in all age groups. Thus, a transpersonal understanding of home care recognizes the human being as an energy field connected to the energy fields of others and to the totality of humanity. The world is open, dynamic, interdependent, fluid, and continually interacting with variables.

A worldview is necessary to highlight the multiple phenomena of the home context and human experience. Home nurses are an instrument of the healing process, facilitating it through their knowledge, actions and being, and strengthening the development of knowledge and understanding of metaparadigm in nursing (nursing, health, person, environment).

Transpersonal home care will be experienced by the person being cared for, caregiver and family in fullness as a development towards personal growth and the expansion of the state of awareness to deeper levels of personal and collective understanding of their physical, mental, emotional, social, spiritual, cultural and environmental dimensions, which constitute a unitary energy field.

## DISCUSSION

In many countries, home nursing care ranges from personal care (e.g., assistance with bathing) to technical nursing care (e.g., wound care), preventive care (e.g., smoking cessation counseling), and psychosocial care (e.g., bereavement support), both on a short-term and long-term basis^(22)^.

However, the authors^(23)^ warn of training that involves the complexity of the context, as each home, family and person is unique, with specific needs.

In some places, skilled and professional nursing care is provided at home, but is not limited to nurses^(24,25)^. Meanwhile, in other settings, care is primarily provided by family members, with teaching and support from hospitals or healthcare providers^(26)^. It is understood as an “alternative modality to the hospital”^(24,27)^ to provide continuity and/or reduce costs, complications and infections^(28)^.

It presents challenges to workers, requiring an understanding of the uniqueness of the space in which the work is carried out, involving aspects that go beyond controlled environments, such as the hospital. It encompasses economic, social and emotional aspects of users and their family, available resources, social support network, hygiene conditions and home safety^(23,28)^.

It tends to be closer and more oriented to users’ singularities and needs, who remain inserted in their life context, and enables effective interrelationships among healthcare professionals, patients and families. It is then possible to seek a unique transpersonal interrelationship between professional caregivers and persons being cared for^(29,30)^. Thus, we enter the intimate, affectionate, emotional space of people, into which healthcare professionals are invited. Therefore, the goal is to offer professional care, developing autonomy through the search for instrumentalization of care, without going beyond ethical and legal competencies^(31)^.

Since it is performed at the place of residence of the person being cared for and their family, it is considered dynamic and unique. Thus, the care provided is unique and based on each patient’s and family’s needs^(24,25,32,33,34)^.

The presence of a social support network, provision of social and economic aspects, participation of family caregivers, health territory management and organization becomes peculiar and important so that care can be performed in home environments^(24,27,33)^.

What precedes the concept of home care is the need for care, which arises from vulnerable situations, requiring preventive and health-promoting care, as well as restorative care for illness, which seeks to control chronic diseases, functional and/or cognitive deficiencies, active aging, practices that, in most cases, precede hospitalization^(24)^.

However, it may also be a preference of patients and family members for care to be provided at the place of residence. Faced with the imminent and new experience of care, family caregivers feel concern and doubts^(35,36)^, requiring professionals to offer emotional support before and during home care provision^(37,38,39)^.

Some factors that are considered antecedents relate to changes in home structure, intersectoral coordination and adaptation of equipment so that care can be provided^(39,40,41)^.

Changes in financial resources precede and permeate the moment of home care, as many family caregivers need to end their employment relationship in order to care for their family member, and, at other times, the family’s breadwinner is the patient^(39,40,41)^.

For healthcare professionals, and especially nurses, to be able to provide home care, prior preparation is necessary with training, skills and competencies, understood as professionals’ ability to be supported by humanistic values with prior spiritual preparation, interpersonal relationships, scientific knowledge, reflective thinking and ethical attitude^(34,35,42,43)^.

Home care, when applied, provides continuity of care, management of hospital beds, reduction of readmissions and, as a consequence, reduction of complications and infections^(44)^. It becomes a preferred care environment for patients and family members, where it provides opportunities for lifestyle habits to be maintained, reinserting the person being cared for into their socio-family environment, offering guidance and support to family caregivers^(30,41,44)^. However, the change in the home physical structure and transfer of costs to families are aspects found both as prior to home care provision, preceding care provision, and as consequences of care provision in this environment^(39,40,41,42)^.

In the same way that it provides a reintegration of patients into the family bond, it generates concern among family caregivers, with doubts and stress, and among the challenges, they develop strategies for carrying out care^(36,45)^.

The consequences of using the concept of home care also affect nursing professionals, since, during the moment of care, effective interrelations occur among healthcare professionals, patients and families^(46)^. Transforming itself through the care relationship^(25)^, the experience generates reflective learning and the invention of new ways of producing care^(25,28,47)^.

Therefore, meeting basic human needs, strengthening beliefs and emotional care allow transpersonal care to deepen the care relationship in home environments^(29,36)^.

The transpersonal care relationship in the home is influenced by nurses’ awareness and intentionality, implying the singularity of the moment. The middle-range nursing theory for transpersonal home care recognizes the human being as an energy field connected to the totality of humanity, promoting personal growth and expansion of awareness.

### Study limitations

Limitations include changes that may occur over time in scientific knowledge, due to the fact that different attributes may occur for the same concepts in their analyses and due to cultural, contextual and social changes. Therefore, the need for theory theoretical and empirical validation is highlighted, recognizing its limitations and proposing adjustments, as necessary.

### Contributions to health, nursing or public policy

This theory aims to contribute to nursing care in home environments, directing care to meet the diverse physiological, psychological, sociocultural, developmental and spiritual needs of the person being cared for, their family members and the unitary energy field.

The challenge and importance of theoretical and epistemological development in nursing are emphasized, hoping that this research will encourage the application of transpersonal care in teaching, research and home care.

## FINAL CONSIDERATIONS

The creation of a middle-range nursing theory for transpersonal home care was constructed through the formulation of interrelated concepts, assumptions and propositions, in an inductive-deductive rhythm and primarily in a deductive manner, derived from Jean Watson’s Theory of Human Caring/Science of Unitary Caring.

The theory described is abstract enough to be applicable to all individuals cared for, caregivers and families in the home in different social, cultural, political and economic contexts. This gives it the characteristic of being mid-range. Its focus is on home care. Thus, it is not characterized in a generalist way as a grand nursing theory. However, it has greater limits than a theory of practice.
