# Supplementary material for: Construction of a middle-range nursing theory for transpersonal home care
Source: Rev Bras Enferm. 2025 Mar 10;78(1):e20240200. doi: 10.1590/0034-7167-2024-0200 (PMC11893068; doi:10.1590/0034-7167-2024-0200)
Supplement: Translation

# Translation: Construção de uma teoria de enfermagem de médio alcance para o cuidado transpessoal domiciliar

**DOI:** 10.1590/0034-7167-2024-0200pt

**Authors:** Luana Tonin, Maria Ribeiro Lacerda, Paula Manuela Jorge Diogo, Marcos Antônio Gomes Brandão, Marilene Loewen Wall, Paulo Roberto Lima Falcão do Vale

**Affiliations:** I Pontíficia Universidade Católica do Paraná. Curitiba, Paraná, Brasil; II Universidade Federal do Paraná. Curitiba, Paraná, Brasil; III Escola Superior de Enfermagem de Lisboa. Lisboa, Portugal; IV Universidade Federal do Rio de Janeiro. Rio de Janeiro, Rio de Janeiro, Brasil; V Universidade Federal do Recôncavo da Bahia. Santo Antônio de Jesus, Bahia, Brasil

**Keywords:** Teorias de Enfermagem, Formação de Conceito, Enfermagem Domiciliar, Serviços de Assistência Domiciliar, Cuidados de Enfermagem

## Abstract

**Objetivos:**

descrever o desenvolvimento de uma teoria de enfermagem de médio alcance para o cuidado transpessoal domiciliar.

**Métodos:**

trata-se de estudo teórico, que utilizou análise de conceito, síntese de afirmação e derivação. Combinou estratégias de dedução teórica com indução, a partir de revisão da literatura e análise de conceito.

**Resultados:**

o conceito central foi derivado do referencial teórico da Ciência do Cuidado Unitário de Jean Watson. A síntese das afirmações entre os conceitos conduziu à elaboração da teoria de médio alcance. Foram construídos 14 pressupostos e dez proposições, além de um diagrama representativo da teoria de médio alcance.

**Considerações Finais:**

a teoria construída é destinada a contribuir para os cuidados domiciliares, abrangendo as necessidades fisiológicas, psicológicas, socioculturais, desenvolvimentais e espirituais. A validação teórica e empírica é sugerida para confirmar, refutar ou renovar os conceitos, pressupostos e proposições da teoria.

## INTRODUÇÃO

O presente estudo propõe a construção de uma teoria de enfermagem de médio alcance voltada para o cuidado transpessoal domiciliar. Em sintonia com a “Agenda de Prioridades de Pesquisa do Ministério da Saúde”, no eixo de programas e políticas de saúde, especialmente no que tange à atenção domiciliar^(1)^, esta pesquisa se alinha com as prioridades identificadas por líderes de enfermagem domiciliar em diversos países^(2)^.

O aumento projetado na demanda por cuidado domiciliar nas próximas décadas, impulsionado pelo envelhecimento da população e mudanças demográficas globais, torna essencial o desenvolvimento de diretrizes baseadas em evidências e a reestruturação dos sistemas de prestação de cuidados de saúde^(3)^. Em contextos como a União Europeia e os Estados Unidos da América, observa-se uma crescente necessidade de atendimento domiciliar, refletindo na evolução das práticas de cuidado e no impacto das condições de saúde da população^(4,5)^.

A pandemia de COVID-19 intensificou ainda mais a importância do cuidado domiciliar como resposta aos desafios epidemiológicos, sociais e econômicos globais^(6,7)^. A eficácia do cuidado domiciliar transcende a abordagem convencional de saúde-doença, incorporando elementos cotidianos, culturais e contextuais^(8,9,10)^.

A Teoria do Cuidado Humano/Ciência do Cuidado Unitário de Jean Watson é destacada por envolver uma abordagem única, enfatizando a importância da experiência vivida, reconhecendo as dimensões mente-corpo-espírito enquanto campo energético pertencente ao universo unitário^(11)^.

Influenciada por princípios humanitários e existencialistas, a referida teoria inclui o cuidado transpessoal, momento de cuidado e elementos do processo *Clinical Caritas Veritas* ^(11)^. Valorizando múltiplas formas de conhecer, a teoria auxilia enfermeiros na estruturação da prática e na relação com o ser cuidado^(12)^.

Na abordagem do cuidado domiciliar, destaca-se a importância da autenticidade e do relacionamento de confiança, honrando a integridade e a dignidade do indivíduo e da família^(11)^. A presença autêntica do enfermeiro permite uma conexão significativa, facilitando a compreensão das necessidades e promovendo o cuidado transpessoal domiciliar.

Refletindo sobre a disciplina de enfermagem, a utilização de teorias contribui para o conhecimento técnico-científico e o pensamento crítico^(12)^. Destaca-se a importância do desenvolvimento contínuo do conhecimento, com teorias de médio alcance, indicando avanço na epistemologia da área^(13)^.

Para Watson^(14)^, Meleis^(15)^, Smith e Liehr^(13)^, as teorias de médio, grande alcance e situações específicas fornecem uma visão evoluída, compartilhada e unitária do mundo da saúde. Cada teoria de médio alcance reflete filosofias que orientam visões abstratas, influenciando o significado da teoria^(13)^.

Walker e Avant^(16)^ descrevem os níveis de teoria como metateoria, grande teoria, teoria de médio alcance e teoria prática. As grandes teorias, como a Teoria do Cuidado Humano/Ciência do Cuidado Unitário^(11)^, são *frameworks* que explicam fenômenos abstratos, enquanto que as teorias de médio alcance são mais circunscritas, aplicando-se a contextos específicos, como o cuidado domiciliar^(13)^.

Diante do exposto, investigar o cuidado domiciliar e propor relações teóricas entre os elementos e conceitos que congregam o fenômeno podem representar uma contribuição para a área da enfermagem. Isso porque o contexto domiciliar e o cuidado necessitam ser discutidos continuamente, pois este ambiente é permeado por instabilidades nas dimensões fisiológica, psicológica, sociocultural, de desenvolvimento e espiritual, apresentando, consequentemente, variâncias regionais e territoriais.

A fim de elucidar a relevância do estudo, destaca-se que investigações que abordam aspectos relacionados ao cuidado domiciliar e *interfaces* com referenciais teóricos são necessárias e atuais. Outro fator a destacar entre os argumentos que sustentam este estudo diz respeito às lacunas na produção científica em relação à temática. Em extensas revisões integrativas realizadas sobre teorias de médio alcance, nenhuma delas aborda as especificidades do cuidado domiciliar^(17,18)^.

Assim, partiu-se do pressuposto de que o cuidado transpessoal é relevante para o cuidado realizado no domicílio, da mesma forma que a Teoria do Cuidado Humano/Ciência do Cuidado Unitário, grande teoria a ser usada para dedução de uma teoria de médio alcance para o cuidado domiciliar.

## OBJETIVOS

Descrever o desenvolvimento de uma teoria de enfermagem de médio alcance para o cuidado transpessoal domiciliar.

## MÉTODOS

### Aspectos éticos

A pesquisa, embora teórica, respeitou os direitos autorais das publicações incluídas. Seguiu a Lei nº 9610/1998, que regulamenta direitos autorais, considerando o oferecimento de obra científica ao público com consentimento do autor como uma publicação.

### Tipo de estudo

Trata-se de estudo teórico, de natureza descritivo-exploratório, com abordagem qualitativa. No desenvolvimento da teoria de médio alcance, as estratégias podem seguir uma orientação indutiva, dedutiva ou conjugar ambas. As estratégias dedutivas baseiam-se em grandes teorias ou modelos teóricos, enquanto que a abordagem indutiva parte dos dados para organizar e construir uma nova teoria^(19)^.

As estratégias propostas por Walker e Avant^(16)^ foram adotadas neste estudo, incluindo análise, síntese e derivação. A escolha desta estratégia visa à construção prioritariamente dedutiva da teoria de médio alcance para o cuidado domiciliar, permitindo, neste caso, a dedução (derivação) teórica da grande teoria de enfermagem (Teoria do Cuidado Humano/Ciência do Cuidado Unitário)^(11)^.

### Procedimentos metodológicos

#### Coleta, organização e análise dos dados

Na primeira etapa de elaboração de teoria de médio alcance, ocorreu a análise de conceito, compreendida nas oito etapas sequenciais, que incluem a seleção do conceito, a determinação dos objetivos da análise, a identificação das utilizações do conceito escolhido, a determinação dos atributos de definição, a identificação de um modelo de caso, a identificação de casos adicionais, a identificação de antecedentes e consequentes, e a definição de indicadores empíricos^(16)^.

A etapa inicial do processo de análise de conceito foi focada no conceito de cuidado domiciliar. A escolha desse conceito é justificada pela inquietação dos pesquisadores. Além disso, há confusão existente na literatura sobre termos relacionados, como assistência domiciliar, atendimento domiciliar e *home care*.

A fase seguinte envolveu a identificação de todas as utilizações do conceito, orientada pela seguinte pergunta: qual a definição do conceito de cuidado domiciliar para a enfermagem?

Os bancos de dados selecionados incluíram BDENF, LILACS, PubMed, *Science Direct*, Oasis Br e CINAHL. Para cada busca, foi empregada a seguinte estratégia: (“*Home care services*” *OR* “*Home Health Nursing*” *OR* “*Home Nursing*”) *AND* “*Nursing Care*”. A busca foi orientada por bibliotecária experiente.

Foram considerados os estudos no período de cinco anos. Os resultados oriundos da estratégia de busca empregada foram importados para o gerenciador de referências *EndNote Web®*. A pré-seleção dos estudos primários foi realizada pela leitura dos títulos e resumos, com posterior seleção para leitura na íntegra. Ambas as etapas foram realizadas por dois pesquisadores, de forma independente e às cegas, a fim de reduzir o viés de seleção dos estudos, sendo discutidas as discordâncias no processo de seleção. Foram excluídos os estudos que se repetiam e que não correspondiam à pergunta norteadora da revisão.

Foram incluídos 59 estudos na análise do conceito. Estes estudos foram minuciosamente examinados, sintetizados e utilizados para compor a análise do fenômeno, identificando conceitos relacionados e embasando a síntese de afirmações e a derivação teórica.

A análise de conceito, ao gerar extenso material, pode comprometer a consistência interna do conceito^(16)^. Para superar isso, utilizou-se o *software* QSR NVivo 10 na organização e análise de dados^(20)^.

Após a seleção do conceito, determinação de objetivos e identificação de utilizações, as etapas subsequentes incluíram a importação de arquivos para o NVivo 10. A leitura do material selecionado foi acompanhada pela criação de memorandos e nós, essenciais para a reflexão e aprofundamento da análise^(20)^.

A etapa para determinar os atributos de definição, também chamados de atributos críticos, envolve identificar palavras ou expressões frequentemente utilizadas pelos autores para descrever as características do conceito de interesse^(16)^. A questão orientadora foi: quais atributos ou características apontados na literatura estão presentes no conceito de cuidado domiciliar? As palavras e expressões selecionadas foram organizadas em um nó correspondente.

Na etapa de casos modelo e adicionais, o objetivo é ilustrar o conceito pesquisado por meio de um exemplo, real ou fictício, que contenha seus atributos definidores. Deve contribuir para elucidar o que o conceito é exatamente e o que não pode ser^(16)^.

São considerados antecedentes as situações, eventos ou fenômenos que precedem o conceito de interesse^(16)^. No intuito de encontrar os antecedentes do conceito sob análise, estabeleceu-se a seguinte questão: que eventos antecedem a existência do conceito de cuidado domiciliar?

Os consequentes do conceito são os eventos ou situações resultantes da sua utilização. Podem coincidir ou não com os atributos definidores^(16)^. Para verificar a presença das consequências relacionadas ao conceito do cuidado domiciliar, foi utilizada a pergunta: o que resultou após a aplicação do conceito de cuidado domiciliar?

Na etapa final da análise do conceito, buscou-se identificar referências empíricas para os atributos definidores do conceito. Referentes empíricos são categorias ou classes de fenômenos observáveis relacionados aos atributos definidores^(16)^. A literatura selecionada foi (re)consultada para determinar se os indicadores empíricos do cuidado domiciliar foram identificados e se existem instrumentos ou medidas correspondentes.

Na segunda etapa da construção da teoria de médio alcance^(16)^, síntese das afirmações dos conceitos, foi realizada a síntese por meio de especificações das relações com base em evidências provenientes dos estudos selecionados e afirmações combinadas com o referencial teórico da Teoria do Cuidado Humano/Ciência do Cuidado Unitário^(11)^.

Na derivação teórica (terceira etapa), o teórico cria uma nova teoria usando analogia com uma teoria existente. Raciocínio por analogia é fundamental para a inovação, permitindo transferência de ideias de um campo para outro. O teórico busca *insight* em áreas relacionadas e redefine estruturas conceituais para formar uma teoria única^(16)^. Neste caso, foram derivados aspectos estruturais da Teoria do Cuidado Humano/Ciência do Cuidado Unitário^(11)^.

Em relação à classificação em termos de objetivos^(15)^, defende-se que a teoria de médio alcance para o cuidado transpessoal domiciliar construída possa ser considerada descritiva, porque se concentra em descrever os fenômenos relacionados ao cuidado de saúde nesse ambiente. Examina as propriedades, circunstâncias e padrões que ocorrem no cuidado domiciliar, ajudando a categorizar e classificar as observações feitas nesse contexto. Embora possa conter elementos preditivos, sua função principal é explicativa, fornecendo uma base para compreender como e porque certos fenômenos ocorrem no cuidado domiciliar e quais são suas possíveis consequências.

## RESULTADOS

Ao caracterizar os estudos analisados, identificou-se que 33 das publicações se originaram dos países da América do Sul, porém a distribuição geográfica foi bem variada, contendo também publicações da Europa (14), Oceania (1), Ásia (5) e América do Norte (4). Dois trabalhos foram desenvolvidos entre parcerias de dois países (Portugal e Brasil), o que significa que a temática interessa a vários locais do mundo.

Com relação à categoria profissional, 50 foram realizados por enfermeiros como autores principais, profissionais da categoria médica (sete), e ainda um geógrafo e um terapeuta ocupacional. No tocante ao ano de publicação dos estudos analisados, o recorte temporal estabelecido foi entre janeiro de 2015 e dezembro de 2019, sendo que 11 datam de 2015, 15, de 2016, 12, de 2017, dez, de 2018, e 11, de 2019.

O conceito de cuidado domiciliar apresenta variações de significado entre países, sendo praticado por enfermeiros em alguns lugares e predominantemente por membros da família em outros. É considerado uma modalidade alternativa ao hospital para continuidade e/ou redução de custos. A prática enfrenta desafios, exigindo compreensão do ambiente, abrangendo aspectos econômicos, sociais, emocionais e éticos. Além disso, destaca-se como uma modalidade mais próxima e orientada às necessidades dos usuários. O cuidado domiciliar envolve o espaço íntimo das pessoas, promovendo uma interrelação transpessoal singular entre profissional de saúde, ser cuidado e família. Praticado no local de residência, é dinâmico, singular e parte das necessidades de cada paciente e família. A presença da rede social de apoio, aspectos sociais e econômicos, participação dos familiares e relações efetivas entre profissionais, pacientes e família são cruciais. O desenvolvimento de um caso modelo exemplifica os atributos^(21)^.

O caso contrário demonstra o oposto do que é definido como cuidado domiciliar, o qual não demonstra uma boa comunicação entre paciente, família e equipe de saúde, não apontando o planejamento das ações do enfermeiro domiciliar, com foco nos aspectos físicos, sociais, culturais e espirituais^(21)^.

O cuidado domiciliar é antecedido pela necessidade decorrente de condições de adoecimento, doenças crônicas, deficiências funcionais, envelhecimento, e pode ser uma preferência do paciente e familiares. Requer suporte emocional e ajustes na estrutura da casa, recursos financeiros e informações adequadas.

Como consequentes, apresentam-se a continuidade do cuidado, o gerenciamento de leitos hospitalares, a redução de complicações e infecções. Além disso, apresenta desafios e apreensões, como alterações na estrutura da casa e transferência de custos, causando estresse e afetando a organização familiar. Causa impacto nos profissionais de saúde e nas interrelações efetivas entre profissional, paciente e família, gerando aprendizado reflexivo.

Os indicadores empíricos no contexto do cuidado domiciliar representam categorias observáveis que evidenciam a ocorrência do conceito. Esses indicadores medem a preparação profissional antes do cuidado, destacando a formação e a qualificação dos profissionais, e registram a transformação do profissional durante a prestação do cuidado.

A participação da rede social de apoio, formal ou informal, é essencial para o sucesso do cuidado domiciliar. As relações efetivas entre quem cuida e quem é cuidado ocorrem por meio da promoção de fortalecimento de crenças e cuidado emocional, atendendo às necessidades físicas, mentais, sociais e espirituais.

### Teoria de enfermagem de médio alcance para o cuidado transpessoal domiciliar

A partir da avaliação dos atributos, dos antecedentes e consequentes descritos neste estudo e do referencial adotado para derivação desta teoria de médio alcance, elaborou-se a seguinte definição para o cuidado domiciliar: cuidado desenvolvido no ambiente do domicílio pela enfermagem e equipe interprofissional, do nascimento à terminalidade, que inclui a interligação entre aquele que cuida e aquele que é cuidado, utilizando o processo de cuidar com vistas ao *healing*. Requer reconhecer as peculiaridades do contexto domiciliar, da família e rede social de apoio com planejamento, conhecimento, habilidades técnico-científicas, respeito às crenças, à cultura e aos valores individuais.

Após definição do cuidado domiciliar, com base na revisão integrativa da literatura desenvolvida, extraíram-se também outros conceitos relacionados ao fenômeno, como os metaparadigmas da enfermagem (saúde, ser humano, ambiente, enfermagem), cuidador, família, contexto domiciliar, gestão, equipe interprofissional, ética no cuidado domiciliar, rede social de apoio, segurança do paciente domiciliar, formação profissional e cuidado transpessoal. Estes conceitos, quando relacionados, contribuem para descrever o cuidado domiciliar.

O procedimento de síntese afirmativa subsidiou a elaboração de 14 pressupostos e dez proposições, apresentados a seguir:

Pressupostos: o cuidado domiciliar está presente em todo o ciclo vital; o ambiente, no qual o cuidado é desenvolvido, é o domicílio, que guarda especificidades de cada estrutura, cultura e contexto; ao realizar o cuidado neste ambiente, o enfermeiro garante a autonomia do outro (ser humano, cuidador e família) ao respeitar suas decisões livres e responsáveis, sendo isso fundamental no exercício do cuidar; para que o cuidado transpessoal domiciliar possa ocorrer, é necessário procurar a circunstância do outro (ser humano, família e rede apoio) (em outras palavras, significa entender as chaves do contexto domiciliar para entender como ele age); a consideração dos fatores sociais e espirituais, valores, contexto domiciliar, crenças e ideais, que cercam a existência humana, será necessária para imergir nas circunstâncias do ser humano e observar como é afetado; ao realizar o cuidado transpessoal domiciliar, cria-se a preservação da identidade do outro, isto é, cuidar de um sujeito de direitos, um ser singular na história, que tem uma identidade esculpida no tempo, a qual o cuidador deve saber respeitar e promover na medida do possível; no processo de cuidar no domicílio, são atenuadas as necessidades vividas pelo ser humano, mas não só as de ordem física, mas também as de ordem psicológica, social e espiritual (para isso, será necessária uma capacidade adequada de ouvir ou receber o outro, bem como uma competência profissional adequada para solucionar essas necessidades); no ato do cuidado transpessoal domiciliar, o preparo do ser que cuida é fundamental. (cuidar só é possível se imaginarmos o que pode acontecer no futuro e quais necessidades irão se manifestar, para responder com empenho e seriedade a essas necessidades e evitar complicações); para cuidar adequadamente do outro, o ser que cuida deve se sentir devidamente cuidado (familiar cuidador e profissional), e, para tanto, o autocuidado é a condição de possibilidade de cuidar do outro; acompanhar o ser humano de forma transpessoal significa também estar atento à saúde mental, entendida não só como ausência de patologias psíquicas, mas também como apropriação das próprias cognições, ideias, teorias, paradigmas e modos de compreensão da realidade; além disso, o acompanhamento no cuidado transpessoal domiciliar pode ser ofertado também aos familiares cuidadores e à rede social de apoio, por meio de uma gestão responsável dos casos, reconhecendo-os, acolhendo-os, integrando-os e fortalecendo a sua energia no cuidado; acompanhar o ser humano, familiar cuidador e rede social de apoio de maneira transpessoal também significa estar atento ao domínio espiritual, por meio da consciência de ser transcendente, conhecimento dos próprios valores e respeito pela diversidade de culturas e religiões; a competência para o cuidado transpessoal domiciliar está baseada na formação a nível teórico, na formação de competências e habilidades práticas, na capacidade moral de ser sensível ao sofrimento e à atenção ao outro; para cuidar de forma transpessoal, deve-se resgatar a visão integral do ser cuidado, indo na contramão em relação à mentalidade contemporânea que percorre o caminho da fragmentação e da superespecialização.

Proposições da teoria de enfermagem de médio alcance para o cuidado transpessoal domiciliar: cuidar de modo transpessoal no domicílio é um cuidado relacional unitário que ilumina a relação enfermeira-pessoa e o ambiente de *healing*; ao cuidar de modo transpessoal no domicílio, é dada atenção ao corpo, à mente e ao espírito; o cuidado de enfermagem transpessoal no domicílio é uma forma especial de ser, saber e fazer, com o objetivo de proteger, valorizar e preservar a dignidade humana; implica compromisso do profissional de enfermagem no cuidado, atendendo para além das necessidades físicas e abrangendo o ser cuidado em suas necessidades psicológicas, sociais e espirituais; somente a partir do estabelecimento de uma relação de confiança adequada com o ser cuidado e familiar cuidador que poderemos fazê-los compartilhar as informações que considerem relevantes sobre os aspectos que lhes dizem respeito ao contexto domiciliar, com o objetivo de ter mais informações e ferramentas para ajudá-los; para conhecer em profundidade os aspectos relacionados às necessidades de ordem superior do ser cuidado, será necessário considerar aspectos sociais e culturais do indivíduo e aprofundar-se na sua história de vida, considerando todos os aspectos que possam afetá-lo; responsabilidade e colaboração profissional são consideradas atitudes indissociáveis, uma vez que um adequado exercício profissional implica colaboração e trabalho em equipe, a fim de, neste caso, obter diferentes visões profissionais para ajudar o ser cuidado e família a atender às suas necessidades de ordem superior e inferior; considera-se que, para cuidar do outro, devemos primeiro estar atentos à nossa espiritualidade, conhecê-la e trabalhá-la previamente, e a partir de nossa própria experiência como seres espirituais, podemos estar mais atentos às necessidades desse campo no ser cuidado; capacidade de comunicação e atitude de cuidado, mostrando uma atitude ativa, reduzindo o desconforto físico e não físico e promovendo o bem-estar espiritual; habilidades de comunicação adequadas nos permitirão explorar e detectar precocemente as necessidades e recursos espirituais do ser cuidado; a consciência de cuidado transpessoal no domicílio irá transcender o tempo, o espaço e a fisicalidade, tornando-se parte do padrão energético complexo da vida de uma pessoa.

Após o conceito do cuidado domiciliar ter sido analisado e as afirmações relacionais terem sido sintetizadas entre os conceitos e o mesmo derivado a partir do referencial teórico de base, a modelagem da teoria foi então realizada e será apresentada a seguir (Figura 1).

**Figura 1:** Diagrama representativo da teoria de enfermagem de médio alcance para o cuidado transpessoal domiciliar, Curitiba, Paraná, Brasil, 2021

Aplicando a analogia, o diagrama apresentado na figura configura a teoria de enfermagem de médio alcance para o cuidado transpessoal domiciliar. Compreende-se que o cuidado domiciliar ocorre em ambientes variados e em todas as faixas etárias. Assim, um entendimento transpessoal domiciliar reconhece o ser humano como um campo de energia conectado aos campos de energia de outros e à totalidade da humanidade. O mundo é aberto, dinâmico, interdependente, fluido e continuamente interage com variáveis.

Uma cosmovisão é necessária para evidenciar os múltiplos fenômenos do contexto domiciliar e da experiência humana. O enfermeiro domiciliar é um instrumento do processo de *healing*, facilitando-o por meio do seu saber, fazer e ser, e fortalecendo o desenvolvimento do conhecimento e a compreensão do metaparadigma em enfermagem (enfermagem, saúde, pessoa, ambiente).

O cuidado transpessoal domiciliar será experienciado pelo ser cuidado, cuidador e família em plenitude como desenvolvimento em direção ao crescimento pessoal e à expansão do estado de consciência para níveis mais profundos de compreensão pessoal e coletiva de suas dimensões físicas, mentais, emocionais, sociais, espirituais, culturais e ambientais, as quais constituem um campo energético unitário.

## DISCUSSÃO

Em muitos países, os cuidados de enfermagem domiciliar variam de cuidados pessoais (por exemplo, assistência com o banho) a cuidados técnicos de enfermagem (por exemplo, cuidados com ferida), cuidados preventivos (por exemplo, orientações para cessação do tabagismo) e cuidados psicossociais (por exemplo, apoio com luto), tanto de forma breve quanto por períodos longos^(22)^.

No entanto, as autoras^(23)^ fazem um alerta para uma formação que envolva a complexidade do contexto, pois cada domicílio, família e pessoa é único, com necessidades específicas.

Em alguns lugares, há prestação de cuidados de enfermagem qualificados e profissionais no domicílio, mas não limitados aos enfermeiros^(24,25)^. Enquanto isso, em outros locais, os cuidados prestados são principalmente realizados por membros da família, com ensino e apoio de hospitais ou prestadores de cuidados de saúde^(26)^. Observa-se o entendimento como “modalidade alternativa ao hospital”^(24,27)^ para dar continuidade e/ou reduzir custos, complicações e infecções^(28)^.

Apresenta desafios aos trabalhadores, sendo necessária a compreensão da singularidade do espaço em que se desenvolve o trabalho, envolvendo aspectos que vão além de ambientes controlados, como o hospital. Abrange aspectos econômicos, sociais e emocionais do usuário e de sua família, recursos disponíveis, rede social de apoio, condições de higiene e segurança da casa^(23,28)^.

Tende a ser mais próximo e orientado às singularidades e necessidades dos usuários, que permanecem inseridos em seu contexto de vida, e possibilita interrelações efetivas entre profissional de saúde, paciente e família. É, então, possível a busca de uma interrelação transpessoal singular entre o cuidador profissional e a pessoa em cuidado^(29,30)^. Assim, adentra-se o espaço íntimo, afetuoso, emotivo das pessoas, no qual o profissional de saúde é convidado. Portanto, a meta é ofertar cuidado profissional, desenvolvendo autonomia por meio da busca pela instrumentalização do cuidar, não devendo extrapolar as competências ético-legais^(31)^.

Por ser desempenhado no local de residência do ser cuidado e família, é considerado dinâmico, peculiar. Assim, os cuidados realizados são singulares e partem da necessidade de cada paciente e família^(24,25,32,33,34)^.

Torna-se peculiar e importante a presença da rede social de apoio, provisão de aspectos sociais e econômicos, participação dos familiares cuidadores, gestão e organização do território em saúde, para que o cuidado possa ser desempenhado no ambiente domiciliar^(24,27,33)^.

O que antecede o conceito de cuidado domiciliar é a necessidade pelo cuidado, que advém de situações vulnerabilizadas, requerendo cuidados preventivos e de promoção da saúde, bem como cuidados restauradores do adoecimento, que buscam o controle das doenças crônicas, deficiências funcionais e/ou cognitivas, envelhecimento ativo, práticas que, na maioria das vezes, precedem uma hospitalização^(24)^.

No entanto, também pode ser uma preferência do paciente e familiares para que o cuidado seja realizado no local de residência. Diante da iminente e nova experiência de cuidados, os familiares cuidadores sentem preocupação e dúvidas^(35,36)^, requerendo que os profissionais ofereçam suporte emocional antes e durante a realização do cuidado domiciliar^(37,38,39)^.

Alguns fatores que são considerados antecedentes dizem respeito às alterações da estrutura da casa, articulação intersetorial e adequação de equipamentos, para que a realização do cuidado possa acontecer^(39,40,41)^.

As alterações nos recursos financeiros antecedem e permeiam o momento do cuidado domiciliar, pois muitos familiares cuidadores possuem a necessidade de cessar o seu vínculo empregatício para poder cuidar do familiar, e, em outros momentos, o próprio provedor de sustento da família é o paciente^(39,40,41)^.

Para que os profissionais de saúde e, sobretudo, os enfermeiros, possam cuidar no domicílio, é necessário preparo anterior com formação, habilidades e competências, compreendidas como a capacidade de o profissional ter sustentação em valores humanísticos com preparo espiritual anterior, relacionamento interpessoal, conhecimento científico, pensamento reflexivo e atitude ética^(34,35,42,43)^.

O cuidado domiciliar, ao ser aplicado, proporciona continuidade do cuidado, gerenciamento de leitos hospitalares, diminuição de reinternações e, como consequência, redução de complicações e infecções^(44)^. Torna-se um ambiente de cuidado de preferência para paciente e familiares, onde oportuniza que os hábitos de vida sejam mantidos, reinserindo o ser cuidado em seu meio sociofamiliar, ofertando orientação e apoio aos familiares cuidadores^(30,41,44)^. Entretanto, a alteração da estrutura física da casa e a transferência de custos para as famílias são aspectos encontrados tanto como anteriores à realização do cuidado no domicílio, antecedendo a realização do cuidado, quanto como consequências da realização do cuidado nesse ambiente^(39,40,41,42)^.

Da mesma forma que proporciona uma reinserção do paciente ao vínculo familiar, gera apreensão aos familiares cuidadores, com dúvidas e estresse, e entre os desafios, desenvolvem estratégias para a realização do cuidado^(36,45)^.

Os consequentes da utilização do conceito do cuidado domiciliar atingem também os profissionais enfermeiros, pois, durante o momento de cuidado, ocorrem inter-relações efetivas entre profissional de saúde, paciente e família^(46)^. Transformando-se por meio da relação do cuidado^(25)^, a experiência gera aprendizado reflexivo e invenção de novas maneiras de produção do cuidado^(25,28,47)^.

Sendo assim, o atendimento às necessidades humanas básicas, fortalecimento de crenças e cuidado emocional permitem que o cuidado transpessoal aprofunde a relação de cuidado no ambiente domiciliar^(29,36)^.

A relação de cuidado transpessoal no domicílio é influenciada pela consciência e intencionalidade do enfermeiro, implicando a singularidade do momento. A teoria de enfermagem de médio alcance para o cuidado transpessoal domiciliar reconhece o ser humano como um campo de energia conectado à totalidade da humanidade, promovendo crescimento pessoal e expansão da consciência.

### Limitações do estudo

Como limitações, tem-se as mudanças, que podem acontecer ao longo do tempo no conhecimento científico, pelo fato de que podem ocorrer atributos diferentes para os mesmos conceitos em suas análises e pelas mudanças culturais, contextuais e sociais. Sendo assim, destaca-se a necessidade de validação teórica e empírica da teoria, reconhecendo suas limitações e propondo ajustes, conforme necessário.

### Contribuições para as áreas da saúde, enfermagem ou políticas públicas

Essa teoria visa contribuir para os cuidados de enfermagem no ambiente domiciliar, direcionando o cuidado para atender às diversas necessidades fisiológicas, psicológicas, socioculturais, de desenvolvimento e espirituais do ser cuidado, familiares e campo energético unitário

Enfatizam-se o desafio e a importância do desenvolvimento teórico e epistemológico na enfermagem, esperando que a presente pesquisa estimule a aplicação do cuidado transpessoal no ensino, pesquisa e assistência domiciliar.

## CONSIDERAÇÕES FINAIS

A criação da teoria de enfermagem de médio alcance para o cuidado transpessoal domiciliar foi construída por meio da formulação de conceitos, pressupostos e proposições interrelacionados, em ritmo indutivo-dedutivo e prioritariamente de maneira dedutiva, derivada da Teoria do Cuidado Humano/Ciência do Cuidado Unitário de Jean Watson.

A teoria descrita é abstrata o suficiente para ser aplicável a todo ser cuidado, cuidador e família no domicílio nos diferentes contextos sociais, culturais, políticos e econômicos. Isso lhe dá a característica de médio alcance. Seu foco é o cuidado domiciliar. Assim, ela não se caracteriza de forma generalista como uma grande teoria de enfermagem. No entanto, tem limite maior do que uma teoria de prática.
